# Reversible Neurological Toxicity Owing to Metronidazole Use

**DOI:** 10.4269/ajtmh.24-0809

**Published:** 2025-04-15

**Authors:** Rahul Bajaj, Anuj Prabhakar, Deba Prasad Dhibar, Harpreet Singh, Vikas Suri, Ashish Bhalla

**Affiliations:** ^1^ Division of Clinical Infectious Diseases, Department of Internal Medicine, Post Graduate Institute of Medical Education and Research, Chandigarh, India;; ^2^ Department of Neuroradiology, Post Graduate Institute of Medical Education and Research, Chandigarh, India

A 55-year-old male was admitted with intermittent fever up to 38.8°C for 2 weeks and right upper quadrant pain for 5 days. On evaluation, the patient was diagnosed with multiple right-lobe liver abscesses. The patient was managed with pigtail drainage, injection of 1 g ceftriaxone twice a day, and 750 mg metronidazole three times a day for 2 weeks. The patient had positive polymerase chain reaction results from purulent fluid and IgG serology for *Entamoeba histolytica*. He was continued on 800 mg oral metronidazole three times daily for 2 weeks. Repeat pigtail drainage was performed for symptom recurrence and increased abscess size; 800 mg metronidazole three times daily was extended for 2 more weeks. Repeated follow-up ultrasound revealed residual abscess; hence, therapy was extended for 2 more weeks. The patient was followed up after a total of 46 days of metronidazole therapy (with a cumulative dosage of 110.4 g) and had dysarthria, gait disturbances, numbness, paresthesias in the hand and lower limbs, and gastrointestinal adverse effects (nausea and vomiting). On neurological examination, the patient had marked ataxia, bilateral intention tremors, wide-based gait, positive Romberg sign, intention tremors, and bilateral hyperreflexia. Blood investigations revealed alanine aminotransaminases of 62 U/L (normal range = 2–41 U/L) and aspartate aminotransferases of 50 U/L (normal range = 2–40 U/L). Nerve conduction study was suggestive of demyelination. Contrast-enhanced magnetic resonance imaging of the brain revealed hyperintensities involving the bilateral dentate nucleus and splenium of the corpus callosum on T2/fluid-attenuated inversion recovery, suggesting metronidazole toxicity (Figure 1A–C). Metronidazole was stopped, and the patient was given oral thiamine, folate, pyridoxine, and vitamin B12 supplementation. The patient’s speech and gait improved within 7 days, with mild residual peripheral neuropathic symptoms at the 8-week follow-up. Repeat imaging 2 months later (Figure 1D–F) revealed complete resolution of the lesions.

**Figure 1.**
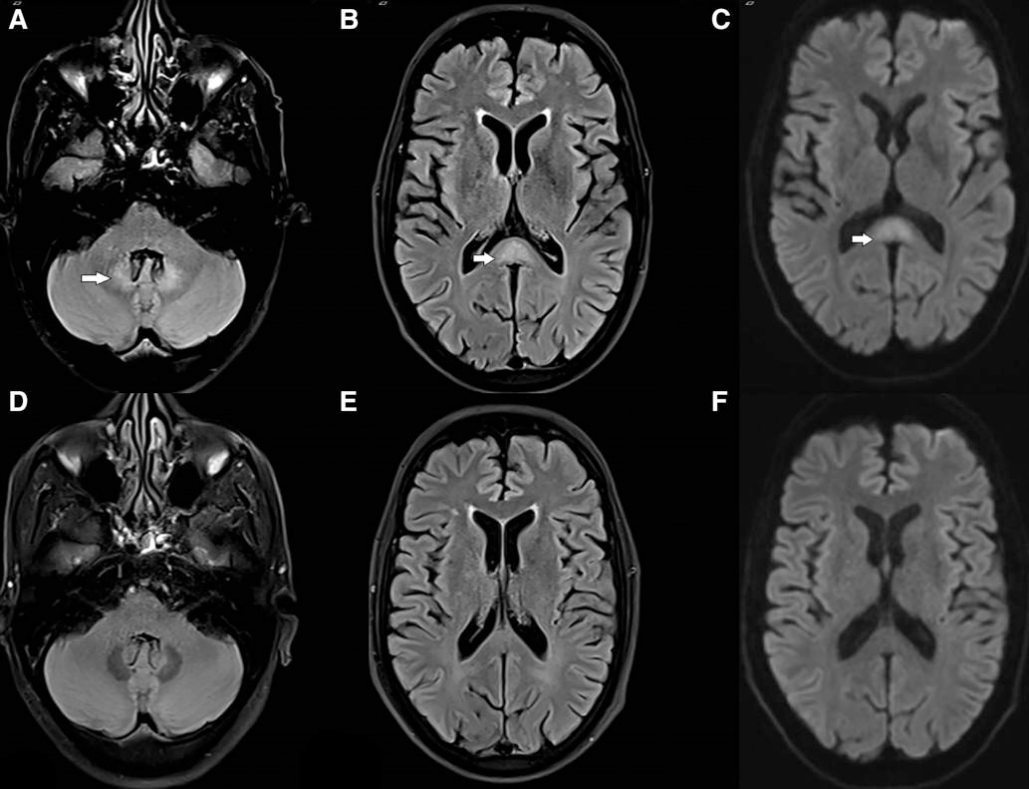
Axial fluid-attenuated inversion recovery (FLAIR) (**A** and **B**) images show symmetrical FLAIR hyperintensities in bilateral dentate nuclei and splenium of corpus callosum with restriction on DWI in the splenium, (**C**) suggestive of metronidazole toxicity (arrow marks). Repeat imaging performed after 2 months revealed complete resolution of the lesions (**D**–**F**).

## DISCUSSION

Metronidazole is commonly used for anaerobic and parasitic infections. Typical side effects include a metallic taste, nausea, and vomiting.^1^ It rarely affects both the central and peripheral nervous systems. Various clinical manifestations include symmetrical painful small fiber involvement along with gait disturbances, dysarthria, and ataxia. Other manifestations may include sensorineural hearing loss, seizures, and encephalopathy.^2^ Central nervous system and peripheral neuropathy may occur at cumulative doses of more than 42 g or for more than 4 weeks.^3^^,^^4^ Complete clinical recovery varies from weeks to months.^1^^,^^5^
